# Cytoprotection against Hypoxic and/or MPP^+^ Injury: Effect of δ–Opioid Receptor Activation on Caspase 3

**DOI:** 10.3390/ijms17081179

**Published:** 2016-08-09

**Authors:** Yuan Xu, Feng Zhi, Naiyuan Shao, Rong Wang, Yilin Yang, Ying Xia

**Affiliations:** 1 Modern Medical Research Center, The Third Affiliated Hospital of Soochow University, Changzhou 213000, Jiangsu, China; 13685262339@163.com (Y.X.); danielzhif@163.com (F.Z.); naiyuanshao@126.com (N.S.); wangrong1949@163.com (R.W.); 2 Department of Neurosurgery, The University of Texas McGovern Medical School, Houston, TX 77030, USA

**Keywords:** Parkinson’s disease, δ-opioid receptor, cytoprotection, hypoxia, MPP^+^, PINK1, caspase 3

## Abstract

The pathological changes of Parkinson’s disease (PD) are, at least partially, associated with the dysregulation of PTEN-induced putative kinase 1 (PINK1) and caspase 3. Since hypoxic and neurotoxic insults are underlying causes of PD, and since δ-opioid receptor (DOR) is neuroprotective against hypoxic/ischemic insults, we sought to determine whether DOR activation could protect the cells from damage induced by hypoxia and/or MPP^+^ by regulating PINK1 and caspase 3 expressions. We exposed PC12 cells to either severe hypoxia (0.5%–1% O_2_) for 24–48 h or to MPP^+^ at different concentrations (0.5, 1, 2 mM) and then detected the levels of PINK1 and cleaved caspase 3. Both hypoxia and MPP^+^ reduced cell viability, progressively suppressed the expression of PINK1 and increased the cleaved caspase 3. DOR activation using UFP-512, effectively protected the cells from hypoxia and/or MPP^+^ induced injury, reversed the reduction in PINK1 protein and significantly attenuated the increase in the cleaved caspase 3. On the other hand, the application of DOR antagonist, naltrindole, greatly decreased cell viability and increased cleaved caspase 3. These findings suggest that DOR is cytoprotective against both hypoxia and MPP^+^ through the regulation of PINK1 and caspase 3 pathways.

## 1. Introduction

Parkinson’s disease (PD) is characterized by the accumulation of cytoplasmic protein inclusions called Lewy bodies in neurons, and the insufficient production of dopamine, which is produced in the substantia nigra of the midbrain [1]. Over the past few decades, many scientists and clinicians around the world have devoted themselves to understanding its pathophysiology, clinical courses and therapies, but until now, there are still limited therapeutic options for PD treatment. Chronic pharmacological therapies that use dopaminergic drugs, are associated with a series of side effects such as L-dopa-induced dyskinesias [2,3] and the risk of tumor formation. Therefore, it is of utmost importance to find novel strategies for PD treatment.

Although the etiopathogenesis of PD is complex, the vulnerability of midbrain dopaminergic neurons to oxidative stress, and environmental neurotoxins affecting the dopamine biosynthetic pathways [4] has long been implicated as potential causes of PD. Furthermore, as the morbidity of PD is greater among older people, it might be associated with age-related conditions such as prolonged ischemia or hypoxia in the brain. There is ample evidence to show that an insufficient blood or oxygen supply to the brain, could attenuate neurons’ resistance to environmental damage, and it can even trigger cell death [5,6,7]. Therefore, hypoxia/ischemia and neurotoxins should also be recognized as critical pathogenic factors that contribute to the development of PD.

One of major breakthroughs in PD research was the discovery of the close association between PINK1 and autosomal recessive familial Parkinson’s disease [8,9]. In healthy mitochondria, PINK1 protects cells from damage-induced mitochondrial dysfunction, oxidative stress and cell apoptosis [10]. However, pathogenic PINK1 mutations in PD lead to a loss-of-function of the PINK1 molecule [11,12,13], leading to depolarization of the mitochondrial membrane potential. Consequently, this induces the mitophagic destruction of mitochondria [14] and then the release of apoptotic signals [15,16]. Like most cells which undergo pathologically mediated cell apoptosis, the midbrain dopaminergic neurons’ death in PD is caspases-dependent [17]. Once the initiator caspases are activated by apoptotic signals, they produce a chain reaction, activating several other executioner caspases, leading to cell death [18,19,20,21,22].

Considering the complexity of the causes, the neuronal signaling involved, and the mechanisms in PD, it is of utmost importance to find new strategies for halting dopaminergic neuron degeneration by protecting neuronal cells against various insults such as hypoxia and neurotoxins. According to our previous work, δ-opioid receptor (DOR) is neuroprotective and actively participates in the control of neuronal survival [23,24,25]. Since hypoxic and neurotoxic insults are underlying causes of PD, and DOR is neuroprotective against hypoxic/ischemic insults, we sought to determine whether DOR activation could protect the cells from damage induced by hypoxia and/or MPP^+^ by regulating PINK1 and caspase 3 expression.

In this work, we used in vitro PD models either in the condition of hypoxia or using MPP^+^ (1-methyl-4-phenyl-pyridinium ion), which is a neurotoxin agent which has been wildly used in animal and cell models by destroying dopaminergic neurons in the substantia nigra in vivo and in vitro [26,27] to mimic PD conditions. Our study was aimed at addressing the following two fundamental questions: Are there any changes in PINK1 and caspase 3 expressions under hypoxic/MPP^+^ stress? Can DOR activation regulate PINK1 and caspase 3 expression and improve cell survival in hypoxia and MPP^+^ models?

## 2. Results

### 2.1. Prolonged Hypoxia and MPP^+^ Stress Caused Severe PC12 Cell Injury

**Figure 1:**
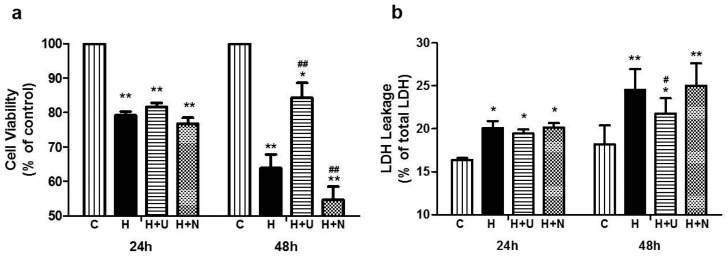
Responses of PC12 cells to DOR activation/inhibition under hypoxia. (**a**) Effects of hypoxia on cell viability. PC12 cells were exposed to hypoxia at 1% O_2_ for 24–48 h, and then the cell viability was measured by an MTT assay; (**b**) Effects of hypoxia on LDH leakage. PC12 cells were exposed to hypoxia at 1% O_2_ for 24–48 h, and then LDH leakage was measured. C: normoxic control. H: hypoxia. H + U: DOR activation with UFP-512 in hypoxic condition. H + N: DOR inhibition with naltrindole in hypoxic conditions. *N* = 3 in each group. * *p* < 0.05, ** *p* < 0.01 vs. control. # *p* < 0.05, ## *p* < 0.01 vs. H. Note that the cell viability was progressively reduced by the increase in hypoxic duration. DOR activation increased cell viability and decreased LDH leakage in hypoxic stress, while DOR inhibition further increased hypoxia induced injury.

Firstly, we used MTT assay to examine the effects of hypoxia and MPP^+^ on PC12 cell viability. The data showed that after hypoxic exposure at 1% O_2_ for 24 h, the cell viability had a significant reduction (79.20% vs. 100% of the control group, *p* < 0.01, *n* = 3; Figure 1a-left panel) and it was further decreased after prolonging the exposure time to 48 h (63.91% vs. 100% of the control level, *p* < 0.01, *n* = 3; Figure 1a-right panel).

**Figure 2:**
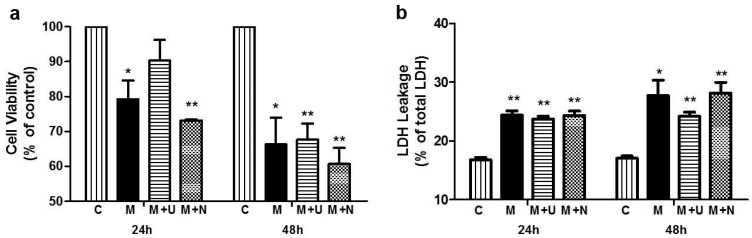
DOR activation protected PC12 cells against MPP^+^ insults. (**a**) Effects of MPP^+^ on cell viability. PC12 cells were exposed to 1.0 mM MPP^+^ for 24–48 h, and then cell viability was measured by an MTT assay; (**b**) Effects of MPP^+^ on LDH leakage. PC12 cells were exposed to 1.0 mM MPP^+^ for 24–48 h, and then LDH leakage was measured. C: normoxic control. M: MPP^+^. M + U: DOR activation with UFP-512 exposed to 1.0 mM MPP^+^. M + N: DOR inhibition with naltrindole exposed to 1.0 mM MPP^+^. *N* = 3 in each group. * *p* < 0.05, ** *p* < 0.01 vs. control. Note that cell viability was progressively reduced by the increase in MPP^+^ exposure duration, DOR activation increased cell viability in the first 24 h and largely prevented LDH leakage after 48 h, while naltrindole further increased the injury induced by MPP^+^.

We also applied MPP^+^ to the PC12 cells to induce parkinsonian injury and chose 1 mM as the working concentration in this work based on our preliminary experiment. MTT assay showed that MPP^+^ induced a similar injury to that of hypoxic stress (Figure 2a). Compared to the control group, MPP^+^ exposure reduced the cell viability by 20.81% (*p* < 0.05, *n* = 3; Figure 2a-left panel) at 24 h and 33.77% at 48 h (*p* < 0.05, *n* = 3; Figure 2a-right panel) after the exposure.

Moreover, we measured LDH (lactate dehydrogenase) leakage in the medium to further validate the effects of hypoxia and MPP^+^ on the PC12 cells. As shown in Figure 1b, LDH leakage progressively increased with the duration of hypoxic exposure, with an increase by 22.42% after 24 h-hypoxia and by 34.92% after 48 h-hypoxia (*p* < 0.01, *n* = 3; Figure 1b). LDH leakage also showed a 45.53% increase (*p* < 0.01 vs. the control, *n* = 3, respectively; Figure 2b-left panel) after MPP^+^ exposure for 24 h and a 62.68% increase (*p* < 0.05 vs. the control, *n* = 3, respectively; Figure 2b-right panel) after MPP^+^ exposure for 48 h.

We also examined cellular morphology using microscope. In the first 24 h of exposure to 1% O_2_ or 1.0 mM MPP^+^, these highly differentiated PC12 cells did not show any appreciable changes in cellular morphology. However, they grew slower in both hypoxic and MPP^+^ conditions as compare the control. After prolonging the exposure duration to 48 h, the numbers of cells sharply decreased, and their shape had major changes, including blebbing and cell shrinkage.

All these information demonstrated that both hypoxic and MPP^+^ stress cause severe injury in PC12 cells.

### 2.2. Both Prolonged Hypoxia and MPP^+^ Insults Increased the Level of Cleaved Caspase 3 with a Reduction of PINK1 Protein

**Figure 3:**
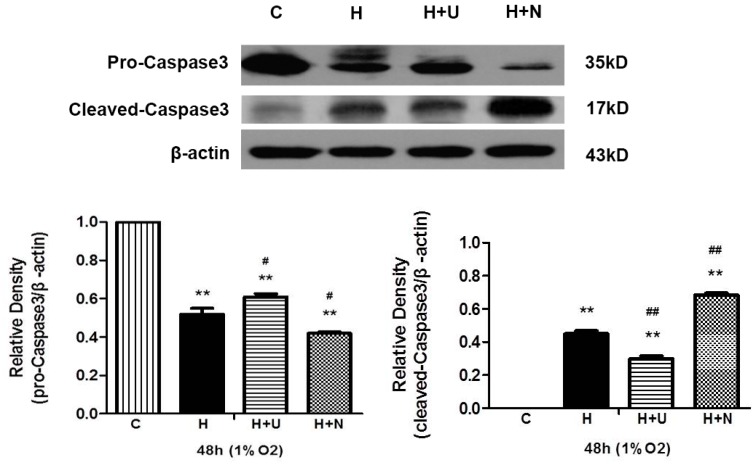
DOR induced attenuation of activated caspase 3 overexpression under hypoxia. Effects of hypoxia on pro-caspase 3 and cleaved caspase 3 expression in PC12 cells. The experiments were conducted under the same conditions described in Figure 1. C: normoxic control. H: hypoxia. H + U: DOR activation with UFP-512 in hypoxic conditions. H + N: DOR inhibition with naltrindole in hypoxic conditions. *N* = 3 for each group. ** *p* < 0.01 vs. control. # *p* < 0.05, ## *p* < 0.01 vs. H. Note that the level of cleaved caspase 3 increased and the level of pro-caspase 3 decreased after 48 h exposure to hypoxia. DOR activation with UFP-512 significantly attenuated the expression level of cleaved caspase 3, whereas naltrindole led to an increase in the level of cleaved caspase 3, and a decrease in that of inactivated caspase 3.

**Figure 4:**
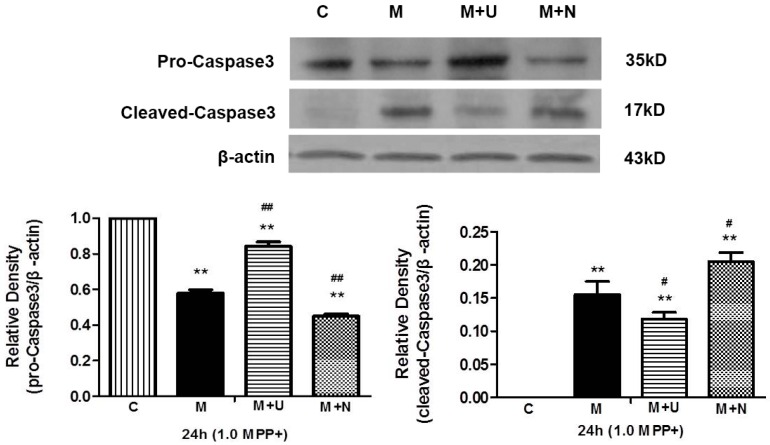
DOR activation attenuated the increase in activated caspase 3 induced by MPP^+^ insults. The experiments were conducted under the same conditions as described in Figure 2. C: normoxic control. M: MPP^+^. M + U: DOR activation with UFP-512 exposed to 1.0 mM MPP^+^. M + N: DOR inhibition with naltrindole exposed to 1.0 mM MPP^+^. *N* = 3 for each group. ** *p* < 0.01 vs. control. # *p* < 0.05, ## *p* < 0.01 vs. M. Note that the level of cleaved caspase 3 increased while the level of pro-caspase 3 decreased after 24-h exposure to MPP^+^. DOR activation with UFP-512 decreased MPP^+^-induced increase in cleaved caspase 3, whereas naltrindole led to an increase in cleaved caspase 3 with a reduction in inactivated caspase 3.

Since caspase 3 activation plays a central role in the execution-phase of cell injury [28], we investigated whether hypoxia and MPP^+^ insults increase the activation of caspase 3 by measuring the changes in the cleaved caspase 3 with apoptotic activity and the full-length caspase 3 protein with no apoptotic activity [18]. As depicted in Figure 3 and Figure 4, the level of full-length pro-caspase 3 (35 kDa) significantly decreased, while the level of cleaved caspase 3 (17 kDa) largely increased after 48 h of hypoxic exposure or 24 h of MPP^+^ exposure.

**Figure 5:**
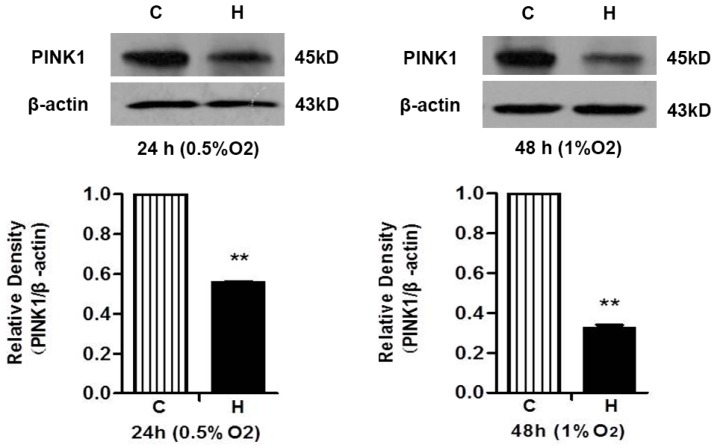
Hypoxia reduced PINK1 protein density in PC12 cells. The effects of hypoxia on PINK1 protein density at different oxygen levels for different durations was analyzed by Western blot. C: normoxic control. H: hypoxia. *N* = 3 for each group. ** *p* < 0.01 vs. control. Note that both hypoxic conditions significantly reduced the level of PINK1 protein, while there was a more significant reduction, when under hypoxia at 1% O_2_ for 48 h.

Since PINK1 is thought to play an important role in protecting nerve cells from parkinsonism by inhibiting caspase 3 action [29], we further investigated if the increase in the activated caspase 3 is associated with a reduction of PINK1 expression in hypoxic and MPP^+^ conditions. As shown in Figure 5, hypoxia at 0.5% of O_2_ for 24 h caused a 44.35% reduction in PINK1 protein (*p* < 0.01, *n* = 3; Figure 5-left panel) and hypoxia at 1% of O_2_ for 48 h led to a more marked decrease (67.53% reduction vs. the control level, *p* < 0.01, *n* = 3; Figure 5-right panel).

**Figure 6:**
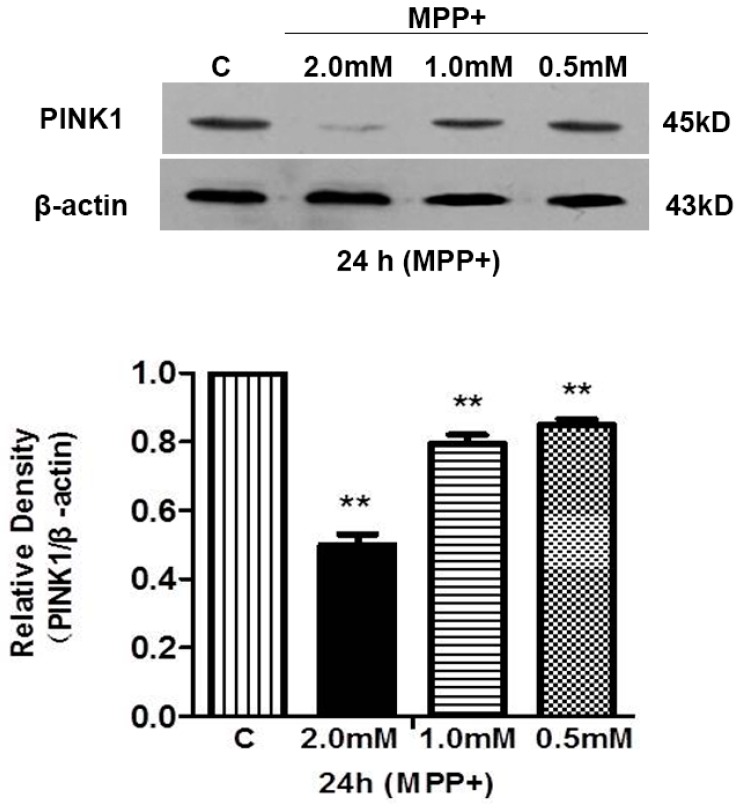
MPP^+^-induced reduction in PINK1 protein of PC12 cells. PINK1 protein was detected using Western blot. C: normoxic control. M: MPP^+^. ** *p* < 0.01 vs. control. Note that MPP^+^ exposure significantly reduced the level of PINK1 protein and this reduction was more marked as the concentration of MPP^+^ increased.

MPP^+^ stress also significantly reduced PINK1 expression (Figure 6). This reduction was dose-dependent in response to MPP^+^ exposure within the concentration range of 0.5 to 2.0 mM. After 24 h-exposure to 0.5 mM of MPP^+^, the relative density of PINK1 protein decreased by 15.10% (*p* < 0.01 vs. the control, *n* = 3; Figure 6). As the MPP^+^ concentration was increased, PINK1 protein density declined more significantly. For example, 24 h-exposure to 2.0 mM MPP^+^ led to a 49.82% reduction in PINK1 signal density (*p* < 0.01 vs. the control, *n* = 3; Figure 6). This data suggests that hypoxia and MPP^+^ insults may negatively regulate PINK1 expression, and thus affect caspase 3 signaling.

### 2.3. DOR Activation Attenuated PC12 Cell Injury Induced by Hypoxic and MPP^+^ Stress

Furthermore, we determined whether DOR activation is protective against hypoxic and MPP^+^ insults.

For the cells under the condition of 24 h-hypoxia with relatively mild injury, the application of DOR agonist UFP-512 (5 μM) or antagonist naltrindole (1 μM) did not cause any significant change in the cell viability. However, after prolonging the hypoxic exposure to 48 h and inducing more severe cell injury, DOR activation largely reversed the reduction of cell viability (from 63.91% in hypoxia alone to 84.30% in hypoxia plus DOR activation, *p* < 0.01, *n* = 3; Figure 2a-right panel), while DOR inhibition further aggravated the hypoxic-induced reduction of cell viability (from 63.91% in hypoxia alone to 54.63% in hypoxia plus DOR inhibition, *p* < 0.01, *n* = 3; Figure 2a-right panel).

DOR activation increased the cell viability by 13.97% (*p* < 0.05, *n* = 3; Figure 2a-left panel) following 24 h-exposure to 1.0 mM MPP^+^. In contrast, DOR inhibition with naltrindole led to a 7.7% reduction in cell viability (*p* < 0.01, *n* = 3; Figure 2a-left panel). On the other hand, the MTT assay did not detect any significant change in MPP^+^-induced injury at 48 h after the administration of UFP-512 or naltrindole though the latter tended to deteriorate the MPP^+^ injury.

With LDH leakage assay, although no significant change was detected at 24 h after the administration of the DOR agonist in hypoxic cells, a significant reduction in LDH leakage (−11.36%, *p* < 0.05 vs. H, *n* = 3; Figure 1b-right panel) was seen at 48 h after the administration of the DOR agonist in hypoxic exposure. Probably because of the sensitive issue in the LDH assessment, DOR activation with UFP-512 or inhibition with naltrindole did not induce any statistically significant changes in LDH leakage after MPP^+^ exposure, although the trends of the changes were consistent with the results of the MTT assay (Figure 2b).

Taken together, DOR activation generally is cytoprotective against hypoxic and MPP^+^ insults, while its inhibition induced an opposite effect.

### 2.4. DOR Activation Upregulated PINK1 Expression in Hypoxia or MPP^+^ Stress

**Figure 7:**
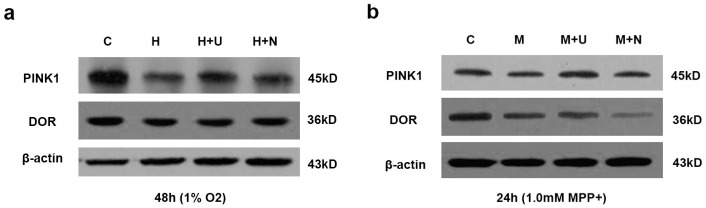
Effect of DOR activation on PINK1 and DOR expression in PC12 cells exposed to hypoxic or MPP^+^ insults. (**a**) PC12 cells were exposed to hypoxia at 1% O_2_ for 48 h. C: normoxic control. H: hypoxia. H + U: DOR activation with UFP-512 in hypoxia. H + N: DOR inhibition with naltrindole in hypoxia. Note that hypoxia at 1% O_2_ for 48 h greatly decreased PINK1 expression, while DOR activation increased the level of PINK1 in the same hypoxic condition. In contrast, DOR expression did not change significantly in hypoxia with/without DOR activation; (**b**) PC12 cells were exposed to 1.0 mM MPP^+^ for 24 h. C: normal control. M: MPP^+^. M + U: DOR activation with UFP-512 in MPP^+^ stress. M + N: DOR inhibition with naltrindole in MPP^+^ stress. Note that DOR activation reversed the reduction of PINK1 expression induced by MPP^+^ insult, and partially attenuated MPP^+^ induced reduction of DOR expression.

Furthermore, we found that DOR activation increased PINK1 expression in hypoxia or MPP^+^ stress. As shown in Figure 7a, the hypoxia-induced reduction in PINK1 expression was greatly reversed by the application of DOR agonist UFP-512. DOR activation also effectively reversed the MPP^+^ induced reduction in PINK1 protein (Figure 7b).

Hypoxia at 1% O_2_ for 48 h did not induce any major change in DOR protein expression. UFP-512 had no appreciable effect on the level of DOR protein (Figure 7a). However, 1.0mM MPP^+^ markedly reduced DOR expression, which was slightly attenuated by DOR activation with UFP-512 (Figure 7b).

### 2.5. DOR Activation Inhibited Hypoxia/MPP^+^-Induced Production of Cleaved Caspase 3

Since caspase 3 is a critical factor in hypoxia and MPP^+^ injury, we further investigated whether DOR activation and inhibition affects caspase 3 signaling. Our data showed that the hypoxia or MPP^+^ induced increase in cleaved caspase 3 was significantly attenuated by DOR activation with more caspase 3 remaining in an inactivated status (full-length protein) (Figure 3 and Figure 4). In contrast, DOR inhibition with naltrindole resulted in a marked increase in cleaved caspase 3, along with a decrease of inactivated caspase 3 under both hypoxic and MPP^+^ conditions.

Our results suggest that DOR signaling may have an inhibitory effect on the transition from inactivated caspase 3 to activated caspase 3.

## 3. Discussion

In this study, we have made several interesting findings regarding the effects of DOR activation on cell survival and PINK1 and caspase 3 expression. Prolonged hypoxia, or a high concentration of MPP^+^ reduced cell viability with a reduction in PINK1 protein and an increase in cleaved caspase 3. DOR activation protected the cells from hypoxia and MPP^+^ injury with an upregulation of PINK1 and downregulation of cleaved caspase 3, while its inhibition induced an opposite effect.

Hypoxia and MPP^+^ stress have been recognized as potential pathogenic factors that contribute to the development of PD [6,7]. Although the specific mechanism underlying neuron death in PD is not yet clearly understood, a large body of evidence strongly supports that mitochondrial dysfunction is evident in the brains of PD patients [30,31]. According to past studies, they induced cell apoptosis through destroying mitochondrial stabilizations [29,30,31]. Moreover, apoptosis has been found to be caspase-dependent [32]. Two classes of caspase-dependent pathways have been identified [33,34]. One of them is dependent on the release of apoptotic factors from mitochondria. In this class, different inducing agents result in a collapse of the mitochondrial transmembrane potential, which brings about the irreversible change in the cell death process [33,34,35].

According to the results of our study, both hypoxia and MPP^+^ insults led to an increase in the expression level of activated caspase 3, along with a decrease in the expression level of precursor forms of caspase 3, suggesting that both hypoxic or MPP^+^-induced cell apoptosis is associated with an increase in caspase 3 activity. Furthermore, we have demonstrated a reduction in PINK1 expression in hypoxic and MPP^+^ conditions. Past studies support the significant role played by PINK1 in neuroprotection, as it protects cells by stabilizing mitochondrial networks through maintaining membrane potential and suppressing mitophagy [36]. It has been proven that in cultured mammalian cells, overexpression of wild-type PINK1 protects cells against apoptosis, whereas small interfering RNA (siRNA)-mediated PINK1 deprivation accelerates apoptotic cell death [29,37]. Since both prolonged hypoxia and MPP^+^ insults reduced the PINK1 protein concentration, we hypothesized that these two cell injury models may share a common mechanism, which is associated with the dysregulation of PINK1 and caspase 3 signaling. As our conjecture, such dysregulation led to a swelling or enlargement of mitochondria due to a loss of membrane maintenance, while mitochondrial dysfunction may be associated with the release of apoptogenic factors, which triggers the conversion of inactive caspases to the active forms, therefore resulting in a reduction in cell viability.

It is interesting to note that DOR activation effectively attenuated cell injury induced by hypoxia and MPP^+^ insults and reversed the reduction in PINK1 and the increase in cleaved caspase 3. Our results strongly suggest that DOR-mediated neuroprotection is closely associated with the rebalance of survival signals via the regulation of PINK1 and caspase 3.

In summary, our findings showed that prolonged hypoxia, and MPP^+^ stress can cause severe cellular injury by decreasing PINK1 expression and thus lead to mitochondrial dysfunction, which is associated with the caspase signaling pathway. DOR activation positively regulated PINK1 expression and negatively affected the transition from inactivated caspase 3 to activated caspase 3, thus attenuating hypoxia or MPP^+^ injury. Our findings sheds a light on DOR’s potential as a promising target that can be applied for clinical neurotherapeutics, especially for Parkinsonism conditions.

## 4. Materials and Methods

### 4.1. Chemicals and Reagents

UFP-512, a specific and potent DOR agonist was produced by our research group. Naltrindole hydrochloride was purchased from Tocris Bioscience (Cat: 0740, Bristol, UK). MPP^+^ (1-methyl-4-phenylpyridinium), MTT powder for cell viability, and fetal bovine serum (FBS) were all purchased form Sigma Chemical Co. (Cat: D048, 15H467, M2128, respectively, St. Louis, MO, USA). LDH cytotoxicity assay kit was purchased from Beyotime Biotechnology (Cat: C0016, Shanghai, China). Dulbecco’s Modified Eagle Medium (DMEM) for cell culture was purchased from Gibco^®^, Thermo Fisher Scientific (Cat: 11995-065, Waltham, MA, USA). Anti-PINK1 antibody was purchased from Novus Biologicals (Cat: BC100-494, Littleton, CO, USA). Anti-β-actin antibody, anti-caspase 3-antibody were all purchased from Cell Signaling Technology (Cat: 4970, 4691, 4060, 9662S, respectively, Danvers, CO, USA).

### 4.2. Cell Cultures and Experimental Groups

Highly differentiated PC-12 cell line was obtained from American Type Culture Collection (Manassas, VA, USA) and maintained in Dulbecco’s Modified Eagle Medium (DMEM), supplemented with 10% FBS. Under normoxic control conditions, the cells were incubated at 37 °C in a humidified atmosphere with 5% CO_2._ Before induction of hypoxia or MPP^+^ Parkinsonism, the cells were cultured in 6-well plates, and were then randomly allocated to normoxia, hypoxia and MPP^+^ groups. For hypoxia, the cells were placed in a hypoxic chamber (Galaxy 48R, New Brun-swick, Edison, NJ, USA) at 37 °C, and its O_2_ levels were kept strictly at 0.5% or 1% by constantly flushing with nitrogen. For mimicking PD conditions, the cells were exposed to 0.5–2.0 mM of MPP^+^. UFP-512 (5 μM) and naltrindole (1 μM) were separately added to the culture mediums before the onset of hypoxia and MPP^+^ exposure. In the normoxic control group, they were added to the culture medium at a similar time point.

There were the following experimental groups:

C: normoxic control.
H: hypoxia.
H + U: DOR activation with UFP-512 (5 μM) in hypoxic condition.
H + N: DOR inhibition with naltrindole (1 μM) in hypoxic condition.
M: MPP^+^.
M + U: DOR activation with UFP-512 (5 μM) under MPP^+^ insult.
M + N: DOR inhibition with naltrindole (1 μM) under MPP^+^ insult.

### 4.3. Cell Viability Assay

Cell viability was measured by a MTT assay. Exponentially growing cells were plated at 6000 cells/well in a 96-well flat-bottom plate, and were allowed to incubate overnight at 37 °C in a humidified incubator with 5% CO_2_. Wells without cells but containing a 200 μL culture medium served as a control for the minimum absorbance. After drug treatment for 24 h, MTT reagent (20 μL/200 μL per well of the 96 well plate) was added, and the cells were incubated another 4 h before measurement. The absorbance was assessed at the wavelength of 490 nm using a microplate reader (BioTek, Winooski, VT, USA).

#### Lactate Dehydrogenase Assessment

Cytotoxicity was quantitatively evaluated by measuring the activity of lactate dehydrogenase (LDH) in the culture medium using a LDH cytotoxicity assay kit. The cells were treated with indicated compounds and incubated for the desired period. Plates containing 10% lactate release reagent and a 200 μL of culture medium without cells were set as maximum control and blank control respectively. After 5 min of centrifugation at 400 g, 120 μL supernatant was transferred to a new 96 well plate and mixed with the provided working solution. These mixtures were incubated at room temperature, protected from light, and for 30 min, the absorbance of each solution was recorded by a microplate reader (BioTek, Winooski, VT, USA) at the wavelength of 490 nm.

% cytotoxicity was calculated as follows:

$$\%\;\text{Cytotoxicity}=\frac{\left[\text{experimental}\;\left(\text{OD}490\right)-\text{blank}\;\left(\text{OD}490\right)\right]\times 100}{\left[\text{maximum LDH release}\;\left(\text{OD}490\right)-\text{blank }\;\left(\text{OD}490\right)\right]}$$

### 4.4. Western Blotting

Cells were lyzed at 4 °C using lysis buffer containing 0.1% protease inhibitor, 1% phosphatase inhibitor and 0.5% 100 mM PMSF (KeyGEN Biotec, Cat: KGP2100, Nanjing, China). The protein concentration was determined using the BCA protein assay kit, and equal amounts of proteins or equal proportions of cell lysates were analyzed using a Western blot. Protein samples were diluted in a 6× sample buffer containing no reducing agent and run in 10% SDS-PAGE. After electrophoresis, proteins were transferred to hydrophobic polyvinylidenedifluorid (PVDF) membranes, and then probed with various mAbs. The binding of mAbs was detected using HRP conjugated secondary antibodies, and visualized using Western Lightening^®^ Chemiluminescence Reagent Plus (Perkin-Elmer, Boston, MA, USA). Quantitation was performed by densitometry using the NIH Image program (Image J).

### 4.5. Statistical Analysis

All data are presented as means ± SEM of at least three independent experiments. Statistical analysis was performed using one-way ANOVA followed by Bonferroni’s multiple comparison test (Prism 5, GraphPad Software, La Jolla, CA, USA).
